# Oral Tolerance Induction to Newly Introduced Allergen is Favored by a Transforming Growth Factor-β-Enriched Formula

**DOI:** 10.3390/nu11092210

**Published:** 2019-09-13

**Authors:** Sébastien Holvoet, Marie Perrot, Nanda de Groot, Guénolée Prioult, Takashi Mikogami, Valérie Verhasselt, Sophie Nutten

**Affiliations:** 1 Nestlé Institute of Health Science, Gastro Intestinal Health Department, 1000 Lausanne, Switzerland; marie.perrot@ymail.com (M.P.); sophie.nutten@nestle.com (S.N.); 2 Nestlé Nutrition, 1814 La Tour de Peilz, Switzerland; ndegroot70@hotmail.com; 3 Nestlé Product Technology Center Nutrition, 3510 Konolfingen, Switzerland; Guenolee.Prioult@rdko.nestle.com; 4 Armor Protéines, 35460 Saint-Brice-en-Coglès, France; takashi.mikogami@gmail.com; 5 University Nice Sophia Antipolis, Hopital de l’Archet, 06200 Nice, France; valerie.verhasselt@uwa.edu.au

**Keywords:** oral tolerance, food allergy, TGF-β, whey protein isolate

## Abstract

Food allergies have become a major healthcare concern, hence preventive efforts to ensure oral tolerance induction to newly introduced antigens are particularly relevant. Given that transforming growth factor-β (TGF-β) plays a key role in immune tolerance, we tested whether an infant formula enriched with TGF-β would improve oral tolerance induction. A partially hydrolyzed whey protein-based formula was enriched with cow’s-milk-derived TGF-β (TGF-β-enriched formula) by adding a specific whey protein isolate (WPI). The manufacturing process was optimized to achieve a concentration of TGF-β within the range of human breast milk concentrations. Protection from allergic sensitization and immune response was assessed in a mouse model. Adult mice received the TGF-β-enriched formula, a control non-enriched formula, or water ad libitum for 13 days before sensitization and suboptimal tolerization to ovalbumin (OVA). When compared to non-tolerized mice, suboptimally-tolerized mice supplemented with the TGF-β-enriched formula showed significantly lower levels of total immunoglobulin-E (IgE) and OVA-specific (IgG1). Mouse mast-cell protease-1 (mMCP-1) and cytokine levels were also significantly decreased in suboptimally-tolerized mice fed the TGF-β-enriched formula. In conclusion, oral supplementation with cow’s-milk-derived TGF-β decreased allergic responses to newly introduced allergens and thus reduced the risk of developing food allergy.

## 1. Introduction

Allergies have become a major public health concern. While the initial upsurge in respiratory allergies (asthma, allergic rhinitis) has now been stabilized, food allergies are emerging as the “second wave” of the allergy epidemic [1]. According to studies based on oral food challenges, up to 10% of children are now affected by food allergies in developed countries [2,3].

The normal response to ingested food is oral tolerance, i.e., a state of active non-inflammatory responsiveness to food antigens [4]. This active immunologic process is mediated by the gut-associated intestinal lymphoid tissue and the generation of regulatory T (Treg) cells [4,5]. A food allergy results from an imbalance between allergen-specific Treg cells and Th2 cells. The dominant Th2 immune response is responsible for IgE class-switching and expansion of allergic effector cells observed in allergic patients [6,7].

Breast milk contains compounds that assist in the development and maturation of the infant immune system [8,9,10]. These include bioactive immunomodulatory factors such as secretory immunoglobulin A, human milk oligosaccharides, polyunsaturated fatty acids, and cytokines [5,11]. Transforming growth factor-β (TGF-β) is one of the predominant cytokines in human breast milk and has potent immunoregulatory properties [12]. In mammals, three isoforms of TGF-β are expressed: TGF-β1, TGF-β2, and TGF-β3. They form a highly homologous group of compounds with 70 to 80% homology in their structure and they are highly conserved (≈100%) between species [13,14]. In human and cow’s milk, the main isoforms are TGF-β1 and TGF-β2, and notable variations in their levels have been reported with TGF-β2 consistently described as the most abundant isoform [15,16,17]. Regarding structure, TGF-β is secreted as a precursor protein [18]. It is formed by an N-terminal signal peptide, a pro-region called latency-associated protein (LAP), and a C-terminal region. The latter corresponds to the mature TGF-β molecule when released after disruption of the non-covalent interaction between LAP and TGF-β [19]. In human and cow’s milk, the majority (90%) of TGF-β is in the latent form [12,20]. It is speculated that the release of mature TGF-β by proteolytic cleavage takes place in the stomach under the influence of the acidic pH, which exposes the intestinal tract to high concentrations of activated TGF-β [21]. Within the immune system, TGF-β is one of the key players in the induction of tolerance. It exerts a critical role in directing the differentiation of naïve CD4+ T cells to CD4+ CD25+ Foxp3+ Treg cells [13,22]. These Treg cells are responsible for controlling potentially autoreactive cells that may induce autoimmunity, and for preventing inappropriate responses to allergens [23]. In an animal model of food allergy, Chung et al. [24] showed that CD4+ CD25+ T cells and TGF-β play complementary roles in the induction of peripheral tolerance to oral antigens and that they act by regulating the expansion of antigen-specific CD4+ T cells. 

Cow’s-milk-based infant formula is offered to infants when breastfeeding is insufficient or not possible. Studies suggested that during the 4–6 first months of life, supplementation with a specific partially hydrolyzed whey formula may reduce the risk of allergic disease (eczema in particular) in non-fully breastfed infants from both at risk and general populations, as compared to cow’s milk formula [25,26,27]. Whereas non-hydrolyzed cow’s-milk-based infant formulas contain variable levels of TGF-β1 and TGF-β2, hydrolyzed formulas contain virtually no TGF-β. Absence of TGF-β in hydrolyzed formulas is likely due to the enzymatic proteolysis step during manufacturing and is responsible for the degradation of the cow’s milk TGF-β [28]. We hypothesized that supplementing hydrolyzed infant formulas with TGF-β to levels similar to those found in human breast milk may be an appealing approach to obtain the beneficial effects of this cytokine toward food allergy prevention.

In this study, we performed in vitro and in vivo experiments to assess whether a partially hydrolyzed whey-based infant formula enriched with cow’s-milk-derived TGF-β could preserve the bioactivity of TGF-β and promote tolerance to newly encountered allergens.

## 2. Materials and Methods

### 2.1. TGF-β-Enriched Formula

A specific basic whey protein isolate (WPI) obtained using ion exchange chromatography from skimmed cow’s milk (Vitalarmor^®^ GF-100, Armor Protéines, Saint-Brice-en-Coglès, France) [29,30] was used as a source of cow’s-milk-derived TGF-β. The WPI was appropriately added or not to a partially hydrolyzed whey-based infant formula during the manufacturing process to obtain the TGF-β-enriched formula and the non-enriched formula (control formula, Nestlé, Konolfingen, Switzerland) containing 3 and 0 µg/L of TGF-β2, respectively.

### 2.2. Quantification of TGF-β1, TGF-β2, and TGF-β3

Quantification of TGF-β1, TGF-β2, and TGF-β3 in WPI was performed using Quantikine enzyme-linked immunosorbent assay (ELISA) kits (R&D Systems, Zug, Switzerland). Briefly, 40 mg of WPI was re-suspended in 20 mL of 0.15 M NaCl (Sigma, Buchs, Switzerland). In order to liberate the mature form of TGF-β detectable using ELISA from a latent complex, samples were acidified via the addition of 1/5 volume of 1.0 M HCl (VWR, Nyon, Switzerland) and incubated for 10 min at room temperature. The pH of the samples was maintained between 1.0 and 2.0 during the process in order to ensure the total liberation of the mature form of TGF-β as described in Lyons et al. [31]. Neutralization was performed via the addition of 1/6 volume of 1.2 M NaOH/0.5 M of acid 4-(2-hydroxyéthyl)-1-pipérazine éthane sulfonique (HEPES) (Sigma). TGF-β in the activated samples was quantified using ELISA within 2 h after the neutralization step following the manufacturer’s instructions. TGF-β in the WPI without the activation step described above was also measured to evaluate the proportion of TGF-β already present in its mature form. To quantify TGF-β2 in the TGF-β-enriched formula, 7.5 g of this formula was re-suspended in 50 mL of 0.15 M NaCl. The activation and quantification steps were performed as described above.

### 2.3. Assessment of TGF-β Bioactivity

A qualitative bioactivity test was set up based on the capacity of TGF-β to inhibit the proliferation of Mv 1 Lu cells (mink vison lung cell line, ATCC Manassas, VA, USA). Cells were plated at 4.0 × 10^4^ cells/mL and incubated in 96-well plates at 37 °C in 5% CO_2_ overnight in MEME medium (minimal essential medium Eagle (Sigma)) supplemented with 10% fetal bovine serum (FBS) (Bioconcept, Allschwil, Switzerland) and 1% penicillin/streptomycin (Sigma). The proliferation of the Mv 1 Lu cell line in the presence of the different isomers of TGF-β was measured after incubation with serial dilutions (1000 to 62.5 pg/mL) of recombinant human TGF-β1 or porcine TGF-β2 (R&D Systems) as positive controls. The WPI and TGF-β-enriched formula were serially diluted and assessed in the same condition. Dilutions of the WPI covered a range of TGF-β2 concentrations from 1000 to 0.0128 pg/mL and those of the TGF-β-enriched formula from 500 to 5.3 µg/L for (corresponding in protein concentration: 6.67 mg to 85 µg protein/L and 3.33 mg to 5.3 µg protein/L, respectively). 

The specificity of the inhibitory activity of the samples was assessed by adding 1 µg/mL of polyclonal anti-human TGF-β1 antibody (R&D Systems) and/or 5 µg/mL of polyclonal anti-porcine TGF-β2 antibody (R&D Systems).

After a 16-h incubation, cells were washed and incubated for 6 h in MEME medium containing ^3^H-thymidine (Perkin Elmer, Schwerzenbach, Switzerland) at a concentration of 5 µCi/mL. Cells were then harvested using a 96-cell harvester (Omnifilter, Perkin Elmer) on a 96-well plate (UniFilter, Perkin Elmer). Plates were dried for 1 h at 50 °C and 40 µL of Microsynt 20 (Perkin Elmer) was added. Cell proliferation was assessed by measuring the β radiation level in counts per minute using a top count NXT instrument (Perkin Elmer). Results were expressed in ^3^H-thymidine incorporation. In some experiments, pretreatment of the samples using the above-mentioned activation step was performed before the addition to cell cultures.

### 2.4. Induction of Allergic Immune Response to OVA in Mice

**Figure 1:**
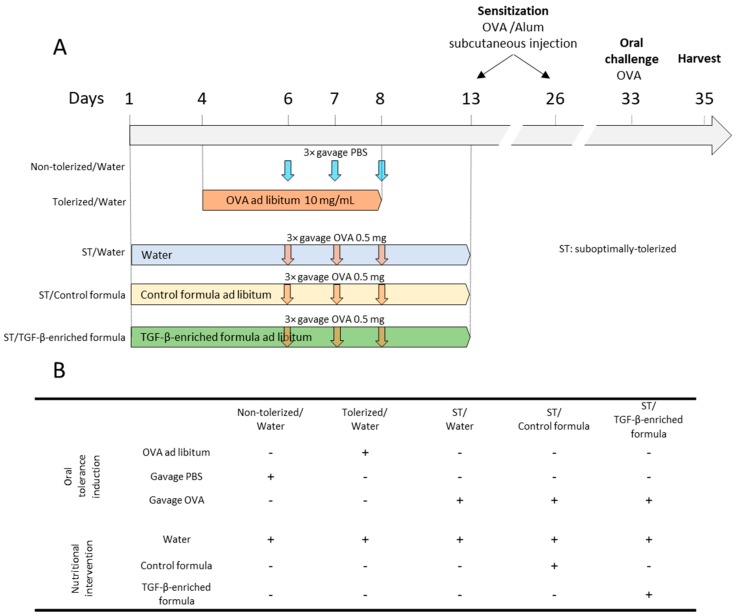
(**A**) Mouse model to assess the impact of a TGF-β-enriched formula on oral tolerance to a newly introduced allergen. A food allergy was induced in 5-week-old female mice using two subcutaneous injections at days 13 and 26 with OVA (100 μg) in alum (1 mg), followed by one oral challenge with OVA (50 mg) via intragastric administration. Oral tolerance was induced with OVA (10 mg/mL) administered ad libitum from day 4 to day 8. Mice receiving a suboptimal-tolerogenic dose of OVA (0.5 mg) using a gavage on days 6, 7, and 8 had free access to either water, non-enriched formula (control formula), or TGF-β-enriched formula from day 1 to day 13. Non-tolerized/water mice only received PBS using a gavage on days 6, 7, and 8. (**B**) represents the different groups receiving (+) or not-receiving (-) OVA for tolerance induction and a nutritional intervention.

The animal study protocol (VD2153.2) was approved by the Service Vétérinaire du Canton de Vaud, Switzerland (Figure 1A). The C57BL/6J CRL mice strain was chosen because it has been successfully used in OVA-induced food allergy and oral tolerance models [32,33,34,35,36]. Briefly, 5-week-old C57BL/6J CRL female mice (Charles River Laboratories, Lyon, France) were sensitized using two subcutaneous injections of 100 µg (Sigma) and 1 mg aluminum hydroxide (Sigma) in 50 µL of phosphate buffer saline (PBS; Sigma) on two separate sites (neck and back) at days 13 and 26. Oral challenge was performed using a gavage with 50 mg OVA in 250 µL of PBS at day 33. At day 35, mice were anaesthetized using isoflurane and euthanized after collecting blood from the abdominal aorta. Axillary/brachial lymph nodes were harvested for further analyses. Mice had free access to food pellets (Kliba 3437, Kliba Nafag, Kaiseraugst, Switzerland) containing wheat, barley, soy, corn, and amino acids as a source of protein. No egg protein was present in the diet.

### 2.5. Oral Tolerance and Oral Supplementation

As shown in Figure 1A, optimal oral tolerance was induced in the above-mentioned food allergy protocol via ad libitum intake of OVA at 10 mg/mL in drinking water from day 4 to day 8 (tolerized/water group) [36]. Suboptimal tolerization was defined as a non-significant decrease in the plasma levels of total IgE and specific IgG1 compared to those found in the non-tolerized/water group. The sub-optimal tolerogenic dose was selected beforehand as the intra-gastric administration of 0.5 mg OVA in 250 μL of PBS on days 6, 7, and 8 according to the dose–response of the above-mentioned plasma IgGs to three intra-gastric administrations of 0.5, 1.0, 2.5, or 5.0 mg OVA. From day 1 to day 13, suboptimally-tolerized (ST) mice were given free access to water (ST/water), the TGF-β-enriched formula (ST/TGF-β-enriched formula) or the control formula (ST/control formula). A description of the different groups tested in this animal model is provided in Figure 1B.

### 2.6. Quantification of Total IgE, and OVA-Specific IgG1 and MCP-1 in Mouse Plasma

Total IgE was quantified in plasma, collected at day 35, using a total mouse IgE kit (BD Bioscience, Allschwil, Switzerland) according to the manufacturer’s protocol. For OVA-specific IgG1, 96-well Nunc MaxiSorp plates (Thermo Fischer Scientific, Zug, Switzerland) were coated with 100 μg/mL OVA (Sigma) overnight at 4°C. Wells were washed with PBS containing 0.05% Tween-20 (Bio-Rad, Reinach, Switzerland) and blocked with PBS-1% bovine serum albumin (Sigma) for 1 h at room temperature. Serially diluted standard monoclonal mouse anti-OVA IgG1 (used as a proxy for Th2 immune response) [37] (Antibody Shop; LucernaChem, Lucerne, Switzerland) and plasma samples were incubated for 2 h at 37 °C, followed by a 2-h incubation with an horseradish peroxidase (HRP)-labelled goat anti-mouse IgG1 antibody (1/5000; Southern Biotech, Allschwil, Switzerland). Plates were developed using tetramethylbenzidine (Thermo Fischer Scientific) and read at 450 nm with a Multiskan Go plate reader (Thermo Fisher Scientific).

Plasma mMCP-1, a mast cell-derived mediator of allergic reaction, was quantified using a mouse MCP-1 ELISA kit (Moredun Scientific, Penicuik, United Kingdom) according to the manufacturer’s instructions.

### 2.7. Isolation and Culture of Axillary and Brachial Lymph Node Cells

Axillary and brachial lymph nodes from each test group were pooled and homogenized with a syringe plunger in a cell strainer (BD Falcon, Meyrin, Switzerland). Cells were centrifuged and washed twice in RPMI medium (Sigma) supplemented with 10% fetal bovine serum (Bioconcept, Paris, France), 1% L-glutamine (Sigma), 1% Penicillin/Streptomycin (Sigma), 0.1% Gentamycin (Sigma), and 50 μM β-mercaptoethanol (Sigma). Cells (3 × 10^5^ cells/well) were cultured in 96-well flat bottom plates (Corning, Meyrin, Switzerland) with or without OVA (1 mg/mL). After 72 h, supernatants from lymph node cultures were frozen at −20 °C until analysis.

### 2.8. Cytokine Quantification in Culture Supernatants

Supernatants from lymph node cultures were collected and mouse interleukine (IL) IL-4, IL-5, IL-10, and Interferon-γ (IFN-γ) were measured using the mouse Th-1/Th-2 multiplex kit (Meso Scale Discovery, Gaithersburg, MD, USA) according to the manufacturer’s instructions. 

### 2.9. Statistical Analyses

**Figure 2:**
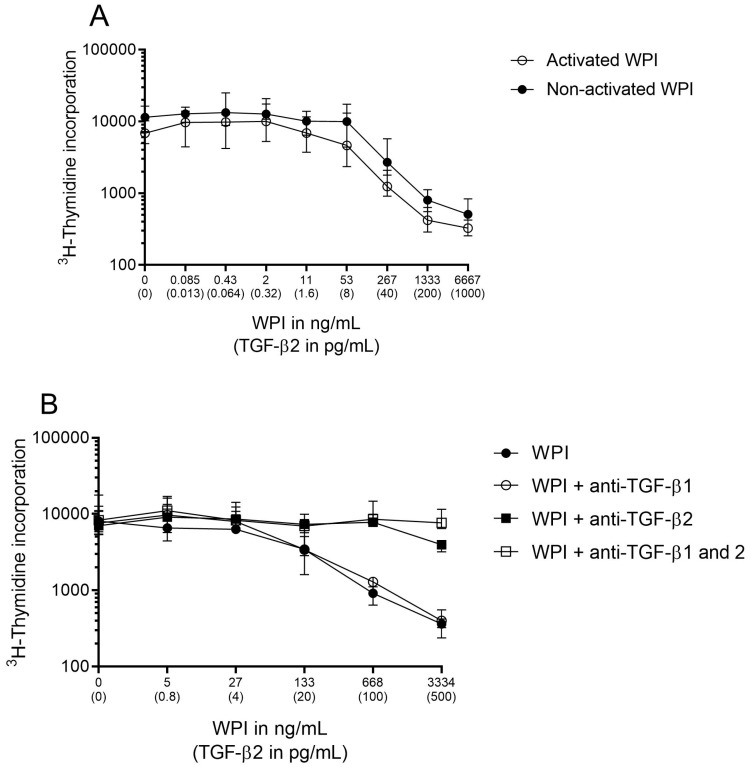

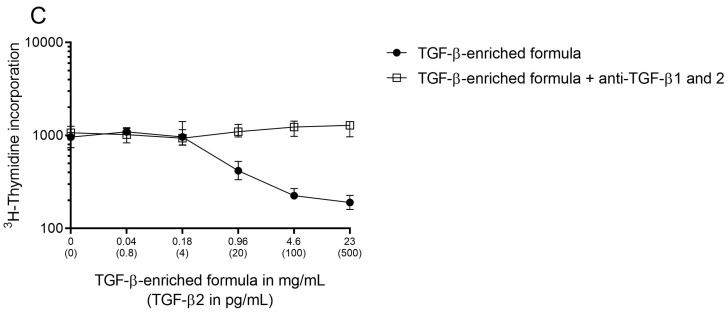
Effect of TGF-β on the inhibition of ^3^H-thymidine incorporation in Mv 1 Lu cell cultures. Mv 1 Lu cells were incubated for 16 h (**A**) with serial dilutions of TGF-β-containing a WPI subjected or not-subjected to an activation step, (**B**) with or without anti-TGF-β1 and/or anti-TGF-β2, or (**C**) with serial dilutions of TGF-β-enriched formula with or without anti-TGF-β1 and anti-TGF-β2. Experiments shown in Figure 2B,C were performed without the activation step. Values are expressed as the median ± interquartile range. Results are representative of one experiment performed with eight replicates (Figure 2A) and one representative experiment out of three performed with four replicates (Figure 2B,C).

For the in vitro data (Figure 2), area under the curves (AUCs) (median) were calculated using the spline method. Exact Wilcoxon tests were performed for all comparisons of interest.

**Figure 3:**
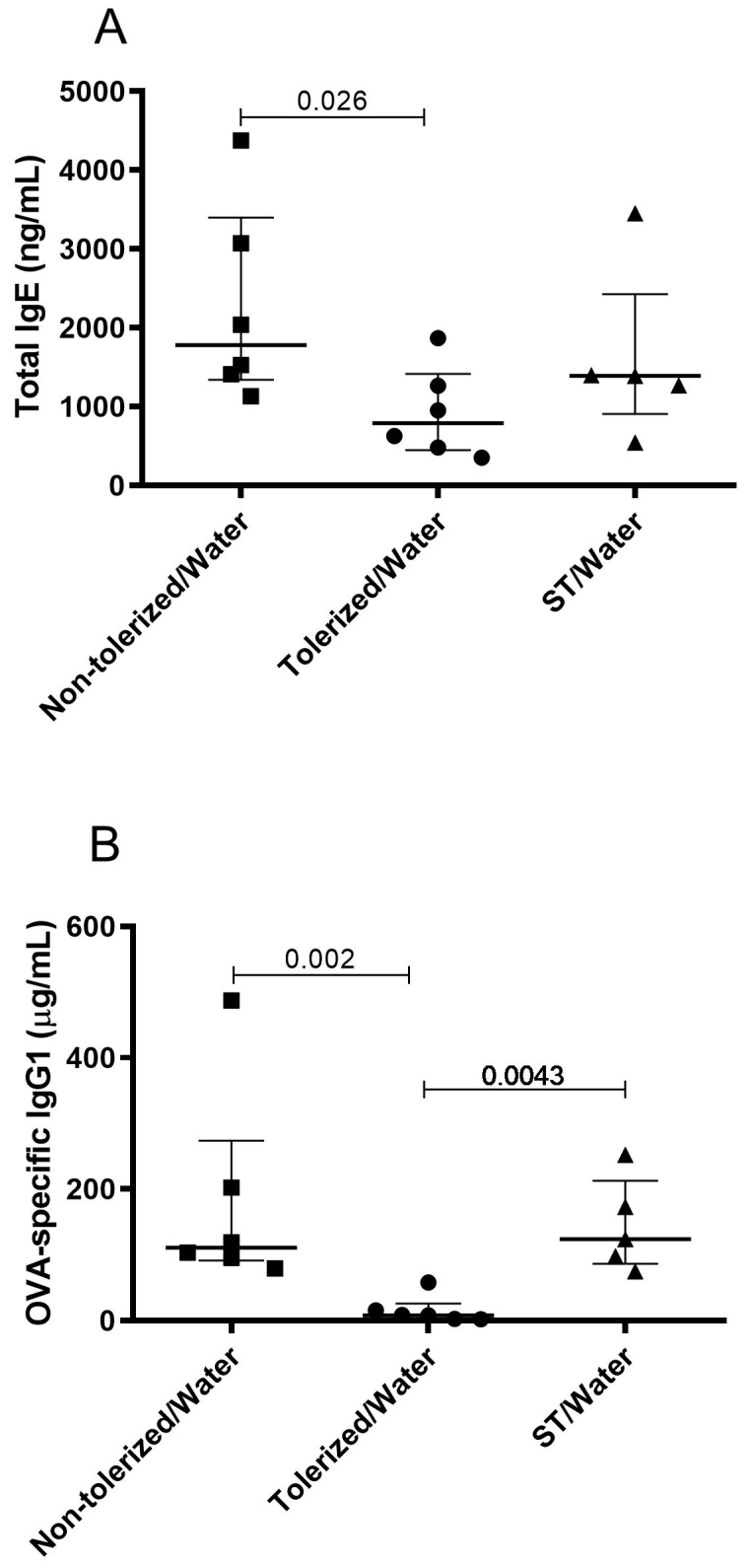
Total IgE (**A**) and OVA-specific IgG1 (**B**) levels in 5-week-old mice subjected to a food allergy protocol. Mice received PBS using a gavage on days 6, 7, and 8 (non-tolerized/water), OVA (10 mg/mL) ad libitum from day 4 to day 8 (tolerized/water), or a sub-tolerogenic dose of OVA (0.5 mg) using a gavage on days 6, 7, and 8 (ST/water). Values represent the median ± interquartile range of six mice for the non-tolerized/water and tolerized/water groups and of five mice for the three ST/water groups. The significant *p*-values (≤0.05) are shown on the graphs.

**Figure 4:**
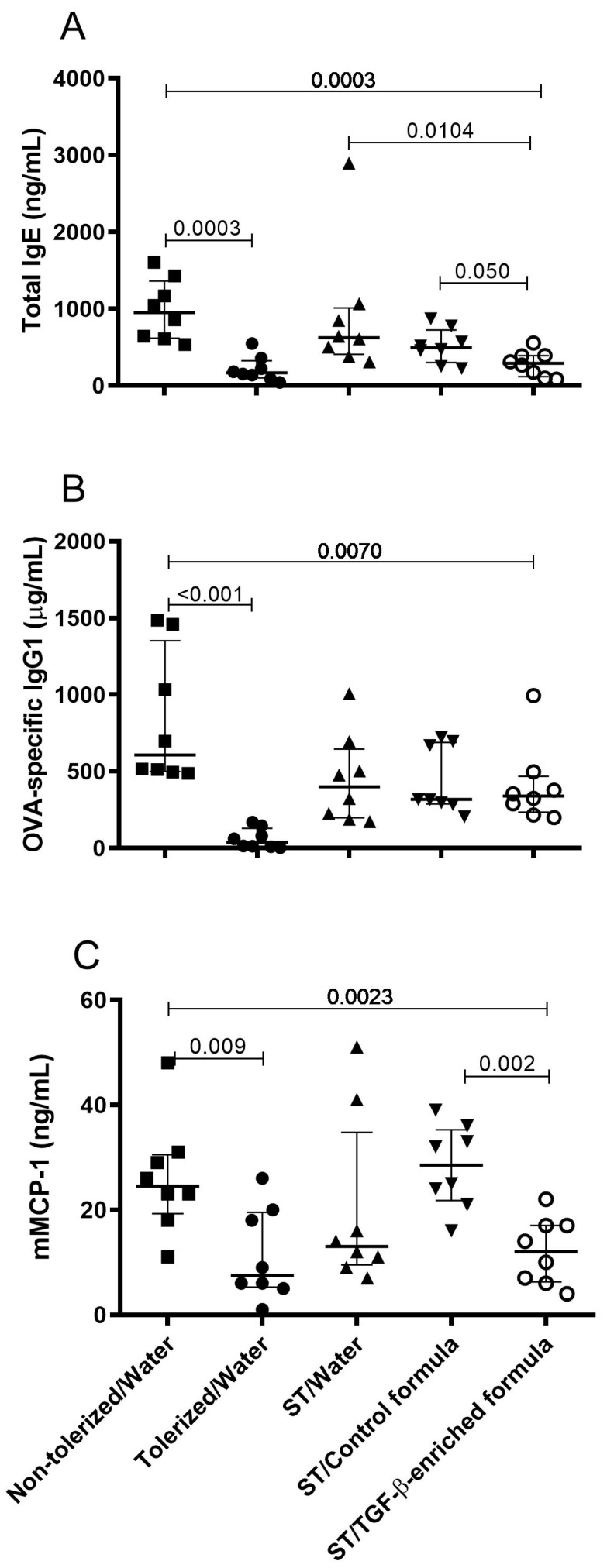
Effect of the oral supplementation of mice with a TGF-β-enriched formula on the protection against sensitization and response to oral exposure to OVA. (**A**) Total IgE, (**B**) OVA-specific IgG1, and (**C**) mMCP1 levels in plasma in 5-week-old mice subjected to a food allergy protocol. Mice received PBS using a gavage on days 6, 7, and 8 (non-tolerized/water), OVA (10 mg/mL) ad libitum from day 4 to day 8 (tolerized/water), or a sub-tolerogenic dose of OVA (0.5 mg) using a gavage on days 6, 7, and 8 (ST/water). ST/water mice had free access to either water, control formula, or TGF-β-enriched formula from day 1 to day 13. Values are expressed as the median ± interquartile range of eight mice per group. Significant *p*-values (≤0.05) are shown on the graphs.

**Figure 5:**
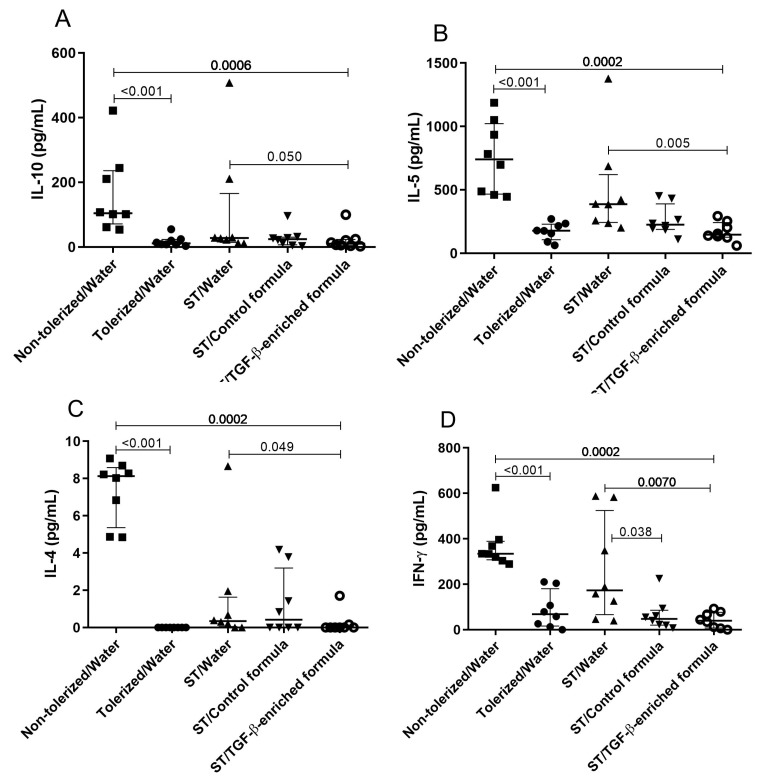
Effects of the TGF-β-enriched formula on cytokine expression in 5-week-old mice subjected to a food allergy protocol. Before sensitization, mice received PBS using gavage on days 6, 7, and 8 (non-tolerized/water), OVA (10 mg/mL) ad libitum from day 4 to day 8 (tolerized/water), or a sub-tolerogenic dose of OVA (0.5 mg) using gavage on days 6, 7, and 8 (ST/water). Suboptimally-tolerized mice had free access to either water (ST/water), control formula (ST/control formula), or TGF-β-enriched formula (ST/TGF-β-enriched formula) from day 1 to day 13. Lymph nodes were harvested from mice after euthanasia at day 35 and pooled by test group. After 72 h of cell culture with OVA (1 mg/mL), the amount of IL-10, IL-5, IL-4, and IFN-γ was determined in the culture supernatants. Results are representative of one experiment out of two. Values are expressed as median ± interquartile range of eight mice per group. Significant *p*-values (≤0.05) are shown on the graphs.

The Kruskal–Wallis test was used first, followed by the Mann–Whitney–Wilcoxon test to adjust the sub-tolerogenic dose (Figure 3), with *n* = 6 mice for the non-tolerized/w ater and tolerized/water groups and *n* = 5 mice for the ST/water group. The exact Wilcoxon test was performed for the oral supplementation experiments (Figure 4 and Figure 5) with *n* = 8 animals in each intervention group. The test was performed with a one-sided test for the comparison of the ST/TGF-β-enriched formula group versus the ST/water group (as this was considered a confirmatory comparison) and a two-sided test for all other comparisons. 

Statistical analyses were conducted using the software R 2.14.1 (R Foundation for Statistical Computing, Vienna, Austria). A *p*-value ≤ 0.05 was considered statistically significant. Data are expressed as median ± interquartile range. 

## 3. Results 

### 3.1. Partially Hydrolyzed Whey Formula Supplemented with TGF-β Containing WPI Contained Bioactive TGF-β

In a first series of experiments, we addressed whether adding a WPI during the manufacturing process of a partially hydrolyzed infant formula could restore TGF-β levels to levels found in human breast milk. The ELISA quantification revealed that the main isoform of TGF-β present in the WPI was TGF-β2 at a concentration of 150 µg/g powder, of which 90 µg/g (60% of the total amount of TGF-β2) were already in the mature form. TGF-β1 was also present at a concentration of 6.9 µg/g powder, of which 3.7 µg/g (54% of total TGF-β1) was in the mature form. No TGF-β3 was detected in the WPI. 

To address whether the cow’s-milk-derived TGF-β in the WPI and the TGF-β-enriched formula preserved its bioactivity, we used the Mv 1 Lu cell line, which is sensitive to both TGF-β1 and TGF-β2, as shown in Supplementary Figure S1. Regardless of the presence or absence of TGF-β activation pretreatment, the WPI inhibited cell proliferation in a dose-dependent manner (similar to recombinant TGF-β2), indicating the bioactivity of cow’s-milk-derived TGF-β and the presence of TGF-β in its active form in the WPI (Figure 2A). To confirm whether the inhibitory effect on cell proliferation observed with the WPI was specific to one isoform of TGF-β, we performed the same experiment in the presence of antibodies that specifically neutralize TGF-β1 or TGF-β2. Adding anti-TGF-β1 antibody to the WPI did not restore cell proliferation (AUC median: 6.58 × 10^5^ ± 9.66 × 10^4^ vs. 5.01 × 10^5^ ± 8.60 × 10^4^; *p* = 0.114). In contrast, cell proliferation was fully restored with anti-TGF-β2 antibody (AUC median: 3.12 × 10^6^ ± 4.01 × 10^5^ vs. 5.01 × 10^5^ ± 8.60 × 10^4^; *p* = 0.029) and with the combination of both antibodies (AUC median: 4.13 × 10^6^ ± 3.29 × 10^5^ vs. 5.01 × 10^5^ ± 8.60 × 10^4^; *p* = 0.029), indicating that TGF-β2 present in the WPI was the main contributor of the observed TGF-β activity (Figure 2B).

The TGF-β-enriched formula also inhibited the Mv 1 Lu cell proliferation in a manner dependent on TGF-β2 concentrations and in a similar fashion to the WPI. This inhibitory effect was totally blocked by a mixture of anti-TGF-β1/2 antibodies (AUC median: 6.40 × 10^5^ ± 2.89 × 10^4^ vs. 1.22 × 10^5^ ± 4.34 × 10^3^; *p* = 0.029) (Figure 2C). The control formula containing virtually no TGF-β showed no effect on cell proliferation. These results showed that the TGF-β found in the WPI and the TGF-β-enriched formula preserved its bioactivity.

### 3.2. TGF-β-Enriched Formula Enhanced the Protection Against Sensitization and Response to an Ovalbumin Challenge

Optimally-tolerized mice (tolerized/water) induced using a free 5-day access to a concentrated OVA solution (10 mg/mL) prior to the subcutaneous sensitization to OVA showed a significant reduction in total IgE and OVA-specific IgG1 as compared to non-tolerized mice (non-tolerized/water) (Figure 3A,B; *p* = 0.026 and 0.002, respectively). In contrast, in suboptimally-tolerized mice (ST/water) receiving a 3-day gavage of a 0.5 mg dose of OVA, the levels of total IgE and OVA-specific IgG1 were comparable with those in non-tolerized mice (non-tolerized/water) (Figure 3A,B; *p* = 0.247 and 1.000, respectively). No OVA-specific IgE was detected in any of the groups (data not shown).

In order to address not only the effect of the TGF-β-containing WPI itself but also its effect in combination with the partially hydrolyzed whey formula on oral tolerance induction, the ST/TGF-β-enriched formula group was compared with the ST/control formula and the ST/water groups, respectively. Following the sensitization and oral challenge to OVA, significant reductions in plasma antibody levels were observed for total IgE in the ST/TGF-β-enriched formula group (Figure 4A; *p* = 0.01 and 0.05 versus the ST/water and the ST/control formula groups, respectively). Total IgE levels were also significantly decreased in the ST/TGF-β-enriched formula group as compared to the non-tolerized/water group (Figure 4A; *p* = 0.0003). OVA-specific IgG1 were significantly decreased in the ST/TGF-β-enriched formula as compared to the non-tolerized/water group (Figure 4B; *p* = 0.007). 

In the present mice model, no symptoms or temperature decrease were observed after the single oral challenge in any of the groups due to a single oral challenge of OVA. Allergic reactions were therefore assessed using the quantification of plasma mMCP-1. Plasma mMCP-1 levels were significantly lower in the ST/TGF-β-enriched formula group than that in the non-tolerized/water group (Figure 4C, *p* = 0.002) or in the ST/control formula group (*p* = 0.002). 

The effect of the TGF-β-enriched formula on cytokine expression was assessed in the lymph nodes draining the sensitization site by measuring IL-10, IL-5, IL-4, and IFN-γ after ex vivo restimulation with OVA. All the cytokine levels in suboptimally-tolerized mice receiving TGF-β-enriched formula (ST/TGF-β-enriched formula) were significantly lower (Figure 5) than those in non-tolerized mice (non-tolerized/water) (*p* = 0.0006, 0.0002, 0.0002, and 0.0002 for IL-10, IL-5, IL-4, and IFN-γ, respectively), or in non-supplemented suboptimally-tolerized mice (ST/water) (*p* = 0.05, 0.005, 0.05, and 0.007 for IL-10, IL-5, IL-4, and IFN-γ, respectively). No significant difference was observed between the ST/TGF-β-enriched formula group and the ST/control group.

## 4. Discussion

TGF-β plays an important regulatory role in the immune response. Its endogenous production is instrumental in regulating mechanisms of oral tolerance and preventing allergy and autoimmunity [38,39,40]. Results from both animal and human studies have shown that the endogenous production of TGF-β in the neonatal intestine is initially very low and increases progressively during weaning [41,42]. Importantly, breast milk is an important exogenous source of TGF-β, which may compensate for this physiological lack of TGF-β in early life [41,43]. Findings from animal models of food allergy supported the idea that oral intake of TGF-β may be critical for oral tolerance induction [43,44,45,46]. In birth cohort studies, there is also some evidence, even though recently controverted [10,47], that high levels of TGF-β in breast milk are correlated with a significant reduction of the risk of allergy in infants and young children [12]. 

Peroni et al. [28] reported that raw unpasteurized cow’s milk contains approximately 0.6 µg/L of TGF-β1 and that boiling and microfiltration significantly reduce the levels of this cytokine; the authors also evidenced undetectable levels of TGF-β1 in different cow’s milk formulas. We detected TGF-β2 in intact cow’s-milk-based formula (from 5.3 µg/L to 27 µg/L) and evidenced undetectable levels in hydrolyzed cow’s-milk-protein-based formula. Consequently, in infants fed hydrolyzed formula, a physiological lack of gut mucosal TGF-β associated with an inadequate exogenous supply may increase the risk for insufficient development and maturation of the mucosal immune system [48]. Importantly, orally administered TGF-β has been found to retain its biologic activity in the gastrointestinal tract and enhance oral tolerance to dietary antigens [44]. Thus, oral supplementation of hydrolyzed formulas with TGF-β to levels comparable with those in breast milk is emerging as a potential strategy to prevent allergic diseases, particularly in infancy. The use of a whey protein fraction containing cow’s-milk-derived TGF-β is a promising approach for TGF-β supplementation. In a murine model, Chen et al. [49] described TGF-β as the main component of a bovine WPI responsible for the reduction in asthma symptoms. Recently, Rekima et al. [50] reported that after weaning, a partially hydrolyzed whey-based formula enriched with TGF-β through the addition of the Vitalarmor^®^ GF-100 WPI may prolong the beneficial effects of breastfeeding on the prevention of food allergy, as compared to a non-enriched formula. Feeding mice with a TGF-β-enriched formula for a prolonged period was shown to be safe as it did not affect mice body weight, intestine growth, nor have an immune suppressive effect [50]. The absence of safety concerns of the use of Vitalarmor^®^ GF-100 in infant formulas was corroborated in 2018 by the European Food Safety Authority Panel on Dietetic Products, Nutrition and Allergies, in the course of its Novel Food authorization [30].

In the present study, we demonstrated the technical feasibility of supplementing a partially hydrolyzed whey protein-based infant formula with bioactive TGF-β similar to levels present in breast milk without modifying the specifications of a partially hydrolyzed formula (<1% of immunoreactive proteins in total proteins). 

A relatively high level of TGF-β2 present in this WPI (150 μg/g) compared to other cow’s milk protein ingredients, as well as an optimized manufacturing process, allowed us to obtain the breast milk TGF-β level in the reconstituted partially hydrolyzed formula with only a small additional amount of intact immunoreactive cow’s milk proteins. In a murine model, we showed that cow’s-milk-derived TGF-β present in the partially hydrolyzed whey formula retained its functional properties by improving the induction of oral tolerance to a newly introduced allergen in sub-tolerogenic conditions. Significant differences were observed between the ST/TGF-β-enriched formula and both the ST/water and the ST/control formula, showing that the TGF-β-enriched formula and the WPI per se could improve oral tolerance to a newly introduced allergen.

The whey basic protein isolate (WPI) used in our study was mainly composed of lactoferrin and lactoperoxidase, where TGF-βs were minor components (approximately 0.016%). However, most of these were heat-sensitive proteins, and as such, were expected to be denatured during the formula manufacturing process; their biological activity was therefore most probably impacted. For example, the presence of lactoperoxydase and its enzymatic activity were assessed in the WPI-enriched formula and could not be detected. As shown in the current study, TGF-β is, on the contrary, resistant to heat-treatment during the formula manufacturing process and its biological activity was well preserved in the formula. However, without deeper investigation, we cannot totally exclude the possibility that any other heat-resistant proteins, such as TGF-β, in the WPI would be involved in the oral tolerance induction observed in vivo. Of note, while not statistically significant, lower levels of IgE were observed in tolerized mice as compared to mice suboptimally-tolerized receiving the TGF-β-enriched formula. This may reflect the fact that the supplementation with the TGF-β-enriched formula in suboptimally-tolerized mice did not fully reach the level of oral tolerance induced by an ad libitum administration of OVA for 5 days. 

While TGF-β in cow’s milk is present mostly in a latent form, TGF-β present in the WPI, thus in the supplemented hydrolyzed formula used in this study, was already mostly in a mature form, which was most likely a consequence of the mild heat-treatment during the manufacturing process. Interestingly, and as reported by others [13], the biological activity of TGF-β is preserved across species. We demonstrated the biological activity of bovine TGF-β in a murine model and can therefore expect similar biological activity in humans, especially since the amino acid sequence of mature TGF-β2 is almost identical in humans and cattle. Oral supplementation of infant formula containing bioactive TGF-β, at a concentration comparable to that in human breast milk, may therefore be a potentially useful approach to prevent allergic diseases in infants who cannot be breastfed.

In conclusion, a partially hydrolyzed whey-protein-based infant formula was enriched with cow’s-milk-derived TGF-β within its concentration range similar to human breast milk by adding a specific WPI. This TGF-β-enriched partially hydrolyzed whey infant formula was shown to improve oral tolerance induction to a newly introduced allergen in mice. This may represent a new strategy to promote oral tolerance and thus potentially reduce the risk of food allergy in infants when breast-feeding is not possible.

## Supplementary Materials

The following are available online at https://www.mdpi.com/2072-6643/11/9/2210/s1, Figure S1: Effect of TGF-β1 and TGF-β2 on the inhibition of ^3^H-thymidine incorporation in Mv 1 Lu cell cultures. Mv 1 Lu cells were exposed to serial dilutions of recombinant TGF-β1 or TGF-β2 with or without anti-TGF-β1 or anti-TGF-β2, respectively. Values are expressed as the median ± interquartile range. Results are representative of one experiment out of three performed with four replicates.
