# Supplementary material for: Oral Tolerance Induction to Newly Introduced Allergen is Favored by a Transforming Growth Factor-β-Enriched Formula
Source: Nutrients. 2019 Sep 13;11(9):2210. doi: 10.3390/nu11092210 (PMC6769637; doi:10.3390/nu11092210)
Supplement: Supplementary file 1 [file nutrients-11-02210-s001.pdf]

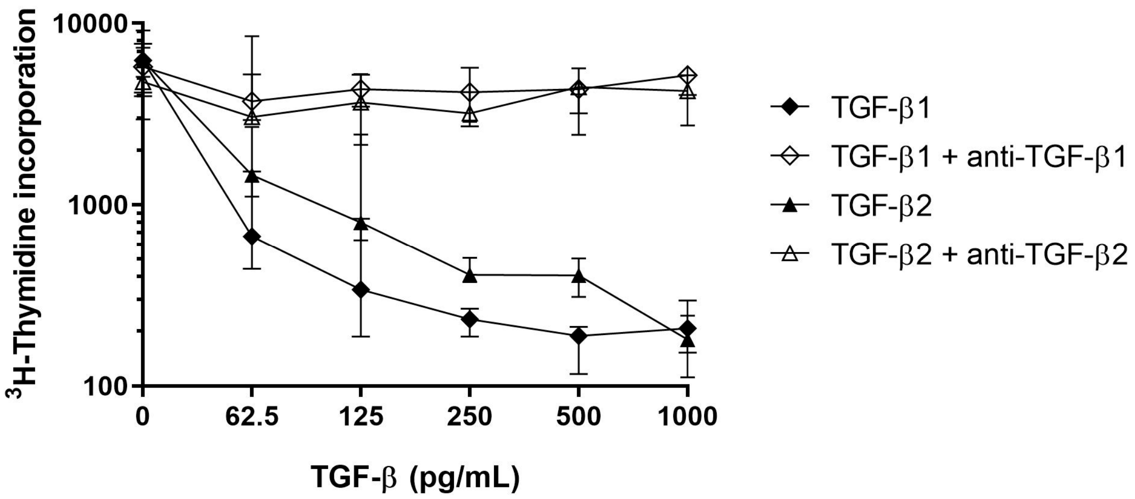

Figure S1. Effect of TGF-β1 and TGF-β2 on the inhibition of ^{3}H-thymidine incorporation in Mv 1 Lu cell cultures. Mv 1 Lu cells were exposed to serial dilutions of recombinant TGF-β1 or TGF-β2 with or without anti-TGF-β1 or anti-TGF-β2, respectively. Values are expressed as the median ± interquartile range. Results are representative of one experiment out of three performed with four replicates.
